# Efficient Preamble Design Technique for Millimeter-Wave Cellular Systems with Beamforming

**DOI:** 10.3390/s16071129

**Published:** 2016-07-21

**Authors:** Dae Geun Han, Yeong Jun Kim, Yong Soo Cho

**Affiliations:** 1 School of Electrical and Electronic Engnineering, Chung-Ang University, Seoul 156-756, Korea; dae450@naver.com; 2 LG Electronics, Seoul 137-893, Korea; yeongjun.kim@lge.com

**Keywords:** millimeter-wave, beam-training, Zadoff-Chu sequence, beamforming, OFDM

## Abstract

The processing time for beam training in millimeter-wave (mmWave) cellular systems can be significantly reduced by a code division multiplexing (CDM)-based technique, where multiple beams are transmitted simultaneously with their corresponding Tx beam IDs (BIDs) in the preamble. However, mmWave cellular systems with CDM-based preambles require a large number of cell IDs (CIDs) and BIDs, and a high computational complexity for CID and BID (CBID) searches. In this paper, a new preamble design technique that can increase the number of CBIDs significantly is proposed, using a preamble sequence constructed by a combination of two Zadoff-Chu (ZC) sequences. An efficient technique for the CBID detection is also described for the proposed preamble. It is shown by simulations using a simple model of an mmWave cellular system that the proposed technique can obtain a significant reduction in the complexity of the CBID detection without a noticeable performance degradation, compared to the previous technique.

## 1. Introduction

Mobile traffic has been increasing markedly owing to the growth of smart devices such as smart phones, tablets, and laptops. By 2020, the mobile traffic volume is expected to increase by more than 1000-fold because of the increase in multimedia and IoT services. In order to support such a considerable increase in the mobile traffic, the 30–300 GHz millimeter-wave (mmWave) frequency band supporting a wide bandwidth is considered as a possible candidate for 5G mobile communication systems with antenna arrays for directional beamforming at both the base station (BS) and the mobile station (MS) [1,2]. The array gain obtained with the directional beamforming extends the coverage of the mmWave systems that suffer from a high path loss owing to a high carrier frequency [3]. Because of the small wavelength of the mmWaves, antenna arrays can be easily installed in the MSs.

Owing to significant costs in the RF chain and the power consumption, analog beamforming is currently preferred over digital beamforming for mmWave communication systems [4]. In mmWave communication systems with analog beamforming, switched beamforming techniques with a set of predefined angles are usually used for transmit-receive (Tx-Rx) beamforming. In these systems, a maximum array gain is obtained when the Tx and Rx beams are perfectly aligned. A Tx-Rx beamforming technique using the 60 GHz unlicensed spectrum has already been standardized for the wireless LAN (IEEE 802.11ad and IEEE 802.15c) to provide a multigigabit-per-second data rate [5,6,7]. In the IEEE 802.11ad standard, a two-level scheme composed of a sector level sweep (SLS) phase and a beam refinement protocol (BRP) phase is adopted for beam training [5]. During the SLS phase, a sector level beam sweep is performed, where the best transmit sector beam is selected with a quasi-omni antenna pattern at the receiver. During the BRP phase, a refined beam selection is carried out with the receive beam at a high resolution to compensate for the imperfections in the quasi-omni antenna pattern. However, in an mmWave cellular system, it is not possible to use the quasi-omni antenna pattern on the receive side because it requires a large transmission range unlike the wireless LANs [8]. Directional beamforming antennas are also necessary at both the BS and the MS to maintain a link budget in the beam-training period. The beam training technique in the IEEE 802.11ad standard cannot be used for cellular communication systems because it is mainly targeted for indoor communication, without a handover capability. The beam training protocol in cellular systems requires a considerable training time and network resources because it needs to select a serving BS with the best beam pair after checking the link qualities of all the possible beam pairs for all the neighbor BSs.

The best beam pair in the mmWave cellular system can be found by using a time-division beam switching (TDBS) during the beam-training period. In the TDBS scheme, single Tx beams are individually transmitted from the BS until all the Tx beams are transmitted. A Tx beam ID (BID) is distinguished by the location of the time slot where the corresponding Tx beam is transmitted. The Rx beam sweep is performed at the MS for each Tx beam for measuring the SNR of every Tx-Rx beam pair. The measurement of the SNRs for all the possible Tx-Rx beam pairs must be performed for all the neighbor BSs to select a serving BS with the best beam pair. The processing time required for beam training in the TDBS increases proportionally to the product of the number of Tx beams, the number of Rx beams, and the number of neighbor BSs. This considerable processing time will create a significant overhead for a moving MS because beam training should be performed periodically for a possible handover or for beam tracking.

In order to reduce the processing time for beam training in mmWave cellular systems, a code division multiplexing (CDM)-based technique was proposed in [9], where multiple beams with their corresponding Tx BIDs are transmitted simultaneously during a beam-training or a beam-tracking period. In [9], the cell ID (CID) as well as the corresponding BID is transmitted in the preamble so that the BID can be detected in a multicell environment. The BID is mapped to a single Zadoff-Chu (ZC) sequence in association with its CID because the ZC sequence is suitable for preamble designs in OFDM systems owing to its good correlation property and low peak-to-average power ratio (PAPR) [10,11,12,13]. However, the computational complexity required for the CID and BID (CBID) detection in [9] increases significantly because the correlation operation required for the CBID detection increases proportionally to the product of the number of CIDs and BIDs. In addition, the number of CIDs in mmWave cellular systems is expected to increase significantly because the cells should be deployed in a denser manner than in the existing systems. Therefore, a new preamble design technique is needed for mmWave cellular systems that can provide a larger number of CIDs and permit a CBID detection with a lower computational complexity.

In this paper, a CDM-based preamble design technique for the mmWave cellular communication systems is proposed using a preamble sequence constructed by a combination of two ZC sequences. It is shown that the proposed preamble design technique can increase the number of CBIDs significantly, compared to the previous technique generated using a single ZC sequence. It is also shown that the proposed technique can reduce the computational complexity in the CBID detection without a noticeable performance degradation, using a specific mapping rule and a parameter selection method. Because of the special structure used in preamble generation, the proposed preamble is shown to have a low PAPR characteristic similar to the one obtained using a single ZC sequence.

The rest of this paper is organized as follows: in Section 2, a preamble design technique using two ZC sequences is proposed for OFDM-based mmWave cellular systems with beamforming. In Section 3, an efficient technique for the CBID detection is described for the proposed preamble. Parameter selection methods are also discussed to avoid the performance degradation caused by a preamble sequence constructed using a combination of two ZC sequences. The performance of the proposed technique is evaluated by computer simulation in Section 4. The conclusions are drawn in Section 5.

## 2. Proposed Preamble Design Technique

The two ZC sequences used to generate a preamble sequence in the proposed technique are given by [14]:

(1) $$Z_{s}^{r,d}\left(m\right)=Z^{r_{s}}\left(m+d_{s}\right)=e^{-j\pi r_{s}\left(m+d_{s}\right)\left(m+d_{s}+1\right)/L},GCD\left(r_{s},L\right)=1$$

where m∈{0,  1, ⋅⋅⋅, L−1},  s∈{0,  1}.

L, s, and GCD denote a prime number representing the length of the ZC sequence, a parameter indicating one of the two different ZC sequences, and the greatest common divisor, respectively. In addition, $r_{s}\in \left\{1,\;\cdot \cdot \cdot ,\;L-1\right\}$ and $d_{s}\in \left\{0,\;1,\;\cdot \cdot \cdot ,\;L-1\right\}$ denote the root index and the cyclic shift of the s-th ZC sequence, respectively. The number of preamble sequences that can be generated by a combination of the root indices and the cyclic shifts of the two ZC sequences, is given by ${\left(L-1\right)}^{2}L^{2}$. Therefore, the number of preamble sequences that can be generated by combining two ZC sequences is considerably larger than the ones generated by a single ZC sequence. For example, the number of available preamble sequences is 3660 for a single ZC sequence with a length of 61 and 864,900 for two ZC sequences with lengths of 31 each. Therefore, the number of preamble sequences that can be generated by combining two ZC sequences with length of 31 is 236 times greater than the ones generated by a single ZC sequence with a length of 61. The proposed preamble is generated by allocating two ZC sequences with lengths, L, in the frequency domain, as follows:

(2) $$X^{r,d}\left(k\right)=\sum_{s=0}^{1}{\tilde{X}}_{s}^{r,d}\left(k\right)e^{-j2\pi ks/N}$$

where ${\tilde{X}}_{s}^{r,d}\left({\left(k+N_{0}\right)}_{\%N}\right)={\tilde{X}}_{s}^{r,d}\left(k\right)$:

$$\begin{array}{l} {\tilde{X}}_{s}^{r,d}\left({\left(m+N_{0}/2-\left\lfloor L/2\right\rfloor \right)}_{\%N_{0}}\right)\\ =\{\begin{array}{cc} Z_{s}^{r,d}\left(m\right), & \;if\;0\leq m\leq L-1\\ 0 & Otherwise \end{array}\\ k\in \left\{0,\;1,\cdot \cdot \cdot ,N-1\right\},N_{0}=N/2 \end{array}$$

**Figure 1:**
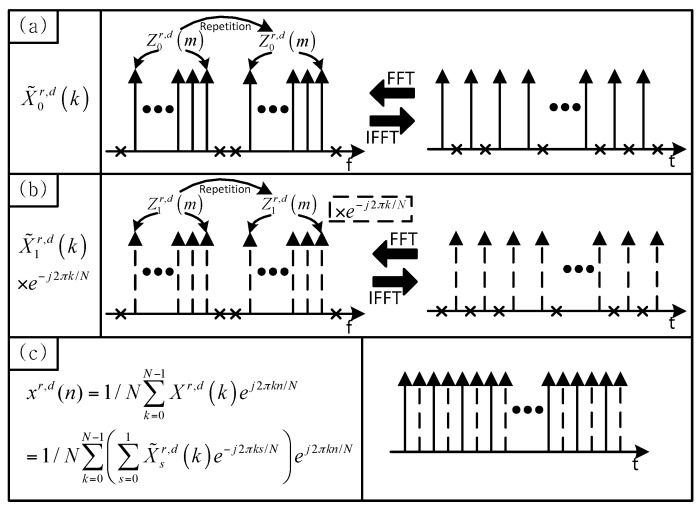
Concept of the preamble generation in the proposed technique.

Here, N denotes the number of subcarriers (FFT size) in an OFDM symbol. Figure 1 shows the concept of the preamble generation in the proposed technique. As shown in Figure 1a,b, ${\tilde{X}}_{0}^{r,d}\left(k\right)$ and ${\tilde{X}}_{1}^{r,d}\left(k\right)$ can be viewed as the two repetitions of $Z_{0}^{r,d}\left(m\right)$ and $Z_{1}^{r,d}\left(m\right)$, respectively. Here, null subcarriers generated by the difference between the FFT size and the sequence length are ignored. As given in Equation (2), $X^{r,d}\left(k\right)$ is the sum of ${\tilde{X}}_{0}^{r,d}\left(k\right)$ and ${\tilde{X}}_{1}^{r,d}\left(k\right)$ multiplied by $e^{-j2\pi k/N}$. The proposed preamble in the time domain can be obtained by performing an IFFT of $X^{r,d}\left(k\right)$, as follows:

(3) $$\begin{array}{l} x^{r,d}(n)=1/N\sum_{k=0}^{N-1}X^{r,d}\left(k\right)e^{j2\pi kn/N}\\ =1/N\sum_{s=0}^{1}\left(\left(1+e^{j\pi \left(n-s\right)}\right)\sum_{k=0}^{N_{0}-1}{\tilde{X}}_{s}^{r,d}\left(k\right)e^{j2\pi k\left(n-s\right)/N}\right) \end{array}$$

where n∈{0,  1,  2, ⋅⋅⋅,  N−1}.

The preamble in Equation (3) can be rewritten as:

(4) $$x^{r,d}\left({\left(2n+q\right)}_{\%N}\right)=2/N\sum_{k=0}^{N_{0}-1}{\tilde{X}}_{s=q}^{r,d}\left(k\right)e^{j2\pi kn/N_{0}}$$

where q∈{0, 1}.

Here, % denotes the modulo operation. The parameter, q, is used to distinguish the even samples (q=0) from the odd samples (q=1). As shown in Figure 1c, $N_{0}$-point FFTs of the even and odd samples of $x^{r,d}\left(n\right)$ are given by ${\tilde{X}}_{0}^{r,d}\left({\left(k\right)}_{\%N_{0}}\right)$ and ${\tilde{X}}_{1}^{r,d}\left({\left(k\right)}_{\%N_{0}}\right)$, respectively. In other words, even and odd samples of $x^{r,d}\left(n\right)$ consist of $Z_{0}^{r,d}\left(m\right)$ and $Z_{1}^{r,d}\left(m\right)$, respectively. Because of this property, the proposed preamble can have a low PAPR characteristic similar to the one obtained using a single ZC sequence.

The correlation property for each sequence (composed of either odd or even samples) can be easily obtained from $x^{r,d}(n)$ [14]. The upper bound for the sum of the correlation values of the two sequences, ${\tilde{C}}^{\bar{r},\bar{d}}$, that will be used for the sequence detection in Section 3, can be derived using the triangular inequality as follows:

(5) $${\tilde{C}}^{\bar{r},\bar{d}}=\{\begin{array}{cc} 1/2\sum_{s=0}^{1}\delta \left({\bar{d}}_{s}\right), & if\;F=0\;(\text{Property }1)\\ 1/\left(2\sqrt{L}\right)\sum_{s=0}^{1}\delta \left({\left\{{\bar{d}}_{s}\right\}}_{\%1}\right), & if\;P\neq 0\;(\text{Property }2)\\ 1/2\left(1/\sqrt{L}+\sum_{s=0}^{1}\delta \left({\bar{d}}_{s}+{\bar{r}}_{s}\right)\right), & if\;F\neq 0,\;P=0\;(\text{Property }3)\; \end{array}$$

where $1/\left(2L\right)\left|\sum_{s=0}^{1}C_{s}^{\bar{r},\bar{d}}\right|\leq {\tilde{C}}^{\bar{r},\bar{d}}=1/\left(2L\right)\sum_{s=0}^{1}\left|C_{s}^{\bar{r},\bar{d}}\right|,\;C_{s}^{\bar{r},\bar{d}}=\sum_{m=0}^{L-1}Z_{s}^{r^{\prime },d^{\prime }}\left(m\right){\left(Z_{s}^{r^{\prime \prime },d^{\prime \prime }}\left(m\right)\right)}^{*}$

$$F=\sum_{s=0}^{1}{\bar{r}}_{s},\;P=\prod_{s=0}^{1}{\bar{r}}_{s},\;{\bar{r}}_{s}=\left|{r^{\prime }}_{s}-{r^{\prime \prime }}_{s}\right|,\;{\bar{d}}_{s}=\left|{d^{\prime }}_{s}-{d^{\prime \prime }}_{s}\right|$$

Here, {${r^{\prime }}_{s}$, ${d^{\prime }}_{s}$} and {${r^{\prime \prime }}_{s}$, ${d^{\prime \prime }}_{s}$} are used to distinguish the parameters of {$r_{s}$, $d_{s}$} for the two different preamble sequences, respectively. δ(⋅) denotes the Kronecker delta function. From Equation (5), it can be seen that if ${\bar{r}}_{s}=0$, ${\tilde{C}}^{\bar{r},\bar{d}}$ becomes one when ${\bar{d}}_{s}=0$ and becomes zero when ${\bar{d}}_{s}>0$. In this case, we can obtain an ideal correlation property. Further, if ${\bar{r}}_{s}>0$, ${\tilde{C}}^{\bar{r},\bar{d}}$ becomes $1/\sqrt{L}$ for all values of ${\bar{d}}_{s}$. In this case, a relatively good correlation property can be obtained. However, when ${\bar{r}}_{s}=0$ and ((${\bar{d}}_{0}>0$ & ${\bar{d}}_{1}=0$) or (${\bar{d}}_{0}=0$ & ${\bar{d}}_{1}>0$)), ${\tilde{C}}^{\bar{r},\bar{d}}$ becomes 1/2 as given in Property 1 of Equation (5). When ((${\bar{d}}_{0}\geq 0$ & ${\bar{d}}_{1}=0$) and (${\bar{r}}_{0}>0$ & ${\bar{r}}_{1}=0$)) or ((${\bar{d}}_{0}=0$ & ${\bar{d}}_{1}\geq 0$) and (${\bar{r}}_{0}=0$ & ${\bar{r}}_{1}>0$)), ${\tilde{C}}^{\bar{r},\bar{d}}$ becomes ($1/2\left(1/\sqrt{L}+1\right)$) as given in Property 3 of Equation (5). These properties (Property 1 and Property 3) can lead to a performance degradation in the sequence detection. In the proposed technique, we select a set of root indices and cyclic shifts such that the case satisfying the condition in Property 1 and Property 3 does not occur or is minimized. The method for selecting the parameters will be described in the next section.

## 3. Efficient CBID Detection Technique for mmWave Cellular Systems

**Figure 2:**
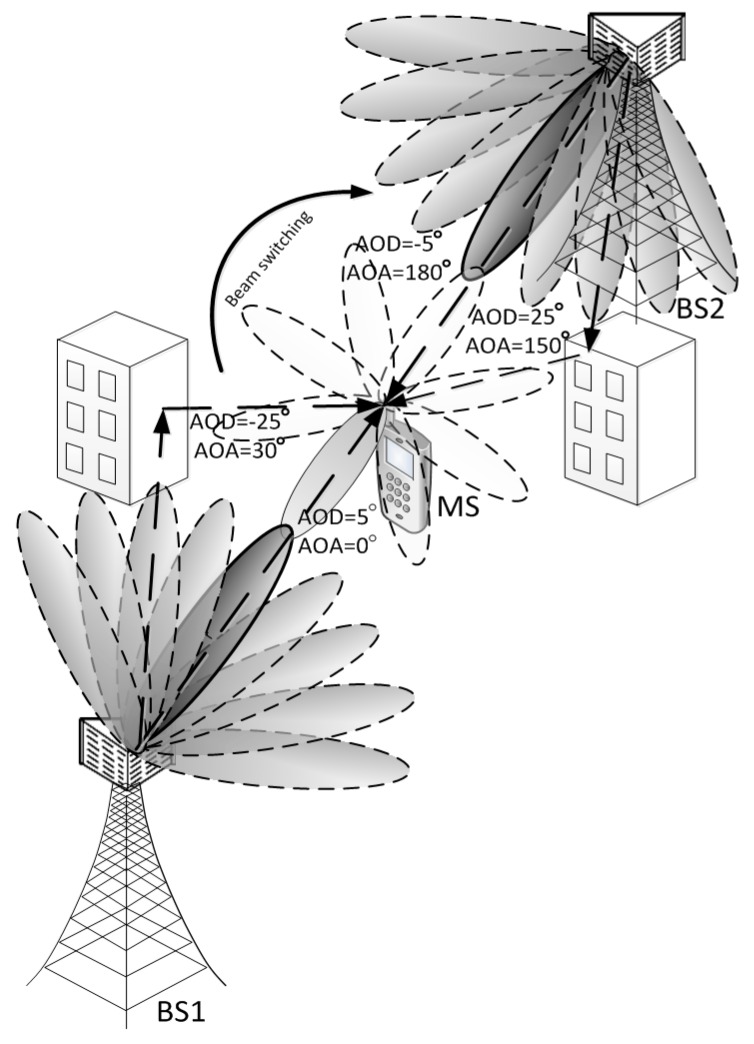
Example of an mmWave cellular system.

In this section, an efficient detection technique that can reduce the computational complexity of the CBID search using the preamble sequence described in the previous section is proposed for mmWave cellular systems. Figure 2 shows an example of an mmWave cellular system with two BSs and one MS. It is assumed that the switched beamforming technique with a set of predefined angles is used at both the BS and the MS. It is also assumed that the BS has multiple antenna arrays, whereas the MS has only one antenna array. That is, multiple beams are transmitted simultaneously from the BS, while a single switched beam is used at the MS. The sidelobes of each beam are omitted in this figure for simplicity. Details on the parameters for the preamble generation, beamforming, and the CBID information will be described in Section 4.

**Figure 3:**
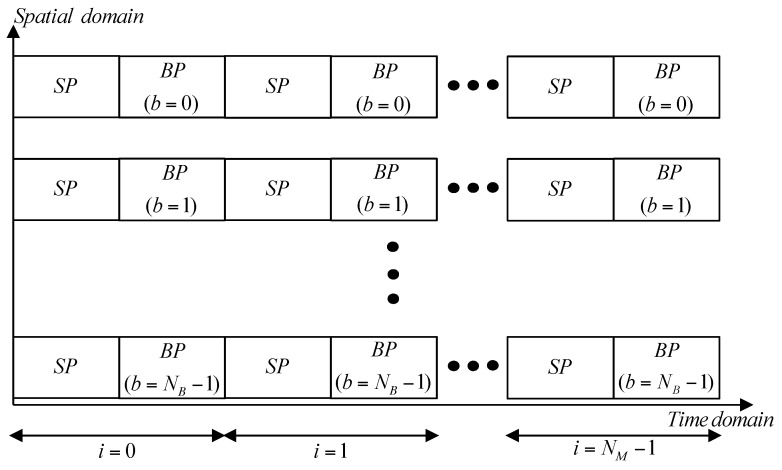
Preamble structure in the proposed technique.

Figure 3 shows the preamble structures used in the proposed technique. Figure 3 shows only the preamble segment, ignoring the data transmission segment in the frame structure. In this figure, $N_{B}$ and $N_{M}$, denote the number of Tx beams (BIDs) and Rx beams, respectively. The variables for the set of BIDs and Rx beam indices are defined as $b\in \left\{0,\;1,\;\cdot \cdot \cdot ,\;N_{B}-1\right\}$ and $i\in \left\{0,\;1,\;\cdot \cdot \cdot ,\;N_{M}-1\;\right\}$, respectively. In Figure 3, ‘*SP*’ represents a synchronization preamble from which the symbol timing offset (STO) and carrier frequency offset (CFO) are estimated. The beam ID associated with the cell ID is acquired from the ‘*BP*’ (BID preamble). In this paper, we assume that the synchronization procedure has been completed with the *SP* using the same approach as in an LTE system with synchronization signals or in a Mobile WiMAX with preambles [12,15]. In this paper, we will focus only on the design of the *BP*. As shown in Figure 3, $N_{B}$ beams are simultaneously transmitted from the BS and the Rx beams are swept over in this period. $N_{B}$ beams are transmitted repeatedly, $N_{M}$ times, until one round of the Rx beam sweep is completed. The Rx beam switching takes place for every 2^nd^ symbol such that the MS can receive a pair of preambles (*SP* and *BP*) for each beam. Note that the processing time for the beam training when the CDM-based scheme is used is $N_{B}$ times smaller than in the case of the TDM-based scheme.

If the CDM-based technique is used for multiple beam transmission in cellular communication systems, the BID as well as the corresponding CID needs to be transmitted in the preamble because the BID needs to be detected in a multicell environment. Therefore, the information (both the CID and the BID) assigned to the beam should be transmitted in the *BP*. A hierarchical preamble design concept is used to carry the BID information as well as the CID information in the *BP*. The information on the CID (c) and BID (b) are mapped to the root indices ($r_{0}$, $r_{1}$) and the cyclic shifts ($d_{0}$, $d_{1}$) given in Equation (1) as follows:

(6) $$Z_{s}^{c,b}\left(m\right)=Z_{s}^{g}\left(m+d_{s}^{c,b}\right)=Z_{s}^{g}\left(m\right)P_{s}^{v,b}\left(m\right)S_{s}^{c,b}$$

where $Z_{s}^{g}\left(m\right)=e^{-j\pi r_{s}^{g}\left(m\right)\left(m+1\right)/L},S_{s}^{c,b}=e^{-j\pi d_{s}^{c,b}\left(d_{s}^{c,b}+1\right)/L}$

$$P_{s}^{v,b}\left(m\right)=e^{-j\pi 2r_{s}^{g}d_{s}^{c,b}m/L}=e^{-j\pi 2{\tilde{d}}_{s}^{v,b}m/L},{\tilde{d}}_{s}^{v,b}=bL_{P}+O_{s}^{v}$$

$$c\in \{0,1,\cdot \cdot \cdot ,N_{C}-1\},\;c=N_{v}g+v,\;N_{g}=\left\lceil N_{c}/N_{v}\right\rceil$$

$$g\in \left\{0,1,\cdot \cdot \cdot ,N_{g}-1\right\},v\in \left\{0,1,\cdot \cdot \cdot ,N_{v}-1\right\}$$

Here, g and v denote the group ID (GID) and sequence ID (SID), respectively. The CID (c) is expressed by a combination of g and v. $N_{C}$, $N_{g}$, and $N_{v}$ denote the number of CIDs, GIDs, and SIDs, respectively. $L_{P}$ denotes the scaling factor for a phase change. $r_{s}^{g}$, $d_{s}^{c,b}$, and $O_{s}^{v}$ denote the root index for the GID (g), the cyclic shift corresponding to the CID (c) and BID (b), and the phase rotation offset for the SID (v), respectively, for the s-th ZC sequence. In Equation (6), $d_{s}^{c,b}$ is selected to satisfy ${\left(r_{s}^{g}d_{s}^{c,b}\right)}_{\%L}={\tilde{d}}_{s}^{v,b}$ so that the phase rotation in $P_{s}^{v,b}\left(m\right)$ does not depend on $r_{s}^{g}$. The GID (g) is mapped to the root index pair of the two ZC sequences, $\left\{r_{0}^{g},r_{1}^{g}\right\}$. The mapping rule for the GID is given by:

(7) $$\left\{r_{0}^{g},r_{1}^{g}\right\}=\left\{{\left({\tilde{g}}^{\prime }\right)}_{\%\left(N_{r}+1\right)}+1,{\left({\tilde{g}}^{\prime }+\left\lfloor {\tilde{g}}^{\prime }/\left(N_{r}+1\right)\right\rfloor \right)}_{\%\left(N_{r}+1\right)}+1\right\}$$

where ${\tilde{g}}^{\prime }=g+\tilde{g}/\left(\tilde{g}+1\right)/2$

$$\tilde{g}=\left\lfloor \left(g+\left\lfloor g/N_{r}\right\rfloor /\left(\left\lfloor g/N_{r}\right\rfloor +1\right)/2\right)/N_{r}\right\rfloor$$

$$r_{s}^{g}\in \left\{1,2,\cdot \cdot \cdot ,N_{r}\right\},\left(N_{r}\leq L-1\right)\&\left(N_{r}\leq N_{g}\right)$$

Here, $N_{r}$ denotes the number of root indices used in the preamble design. An example of Equation (7) when $N_{g}=12$ and ($N_{r}=12$, $N_{r}=7$, $N_{r}=5$) is given in Table 1. In this table, the root index pairs corresponding to the GIDs are listed. For example, 12 root indices are used when $N_{r}=12$, whereas five root indices are used when $N_{r}=5$.

As given in Equation (6), v is mapped to a pair of phase rotation offsets, $\left\{O_{0}^{v},O_{1}^{v}\right\}$. Therefore, c is mapped to a combination of $\left\{r_{0}^{g},r_{1}^{g}\right\}$ and $\left\{O_{0}^{v},O_{1}^{v}\right\}$. When w is defined as $\left\lceil N_{g}/N_{r}\right\rceil$, $N_{C}$ can be expressed by $w\left(N_{r}-\left(w-1\right)/2\right)N_{v}$. The maximum value of $N_{C}$ is obtained when $N_{r}=L-1$ and is given by $w\left(L-\left(w+1\right)/2\right)N_{v}$. Therefore, the proposed technique can increase the number of available CIDs compared to the previous technique with a single ZC sequence. For example, when the length of a ZC sequence is 1021, the number of available CIDs is 1020 in the previous technique and 6084 in the proposed technique, when L, w, and $N_{v}$ are set to 509, 3, and 4, respectively. The number of CIDs in the proposed technique is six times larger than the one in the previous technique. The values of ${\tilde{d}}_{s}^{v,b}$ are selected such that the conditions ${\tilde{d}}_{s}^{v^{\prime },b^{\prime }}\neq {\tilde{d}}_{s}^{v^{\prime },b^{\prime \prime }\neq b^{\prime }}$ and ${\tilde{d}}_{s}^{v^{\prime },b^{\prime }}\neq {\tilde{d}}_{s}^{v^{\prime \prime }\neq v^{\prime },b^{\prime \prime }}$ are satisfied for two different preambles with $\left(v^{\prime },b^{\prime }\right)$ and $\left(v^{\prime \prime },b^{\prime \prime }\right)$. Here, $0\leq v^{\prime },v^{\prime \prime }\leq N_{v}-1$ and $0\leq b^{\prime },b^{\prime \prime }\leq N_{B}-1$. This condition is required to remove the poor correlation properties (Property 1 and 3) in Equation (5). Note that the correlation property of the proposed preamble can be changed depending on the value of $N_{r}$. As $N_{r}$ decreases, the number of side peaks satisfying the condition in Property 3 increases. The maximum number of side peaks is given by 2(w−1). In the example for the GID mapping rule in Table 1(b/c), the maximum number of side peaks satisfying the condition in Property 3 is two/four because the value of w is two/three. For example, the root index 2/3 appears twice/four times for different GIDs, producing high correlation values (side peaks). Thus, as $N_{r}$ decreases, the number of side peaks producing high correlation values at incorrect positions can increase. However, the computation complexity for the CBID detection is reduced as $N_{r}$ decreases because the number of available root indices decreases.

In the CBID detection, it is assumed that $N_{0}$-point FFT is used. However, because the length of the ZC sequence, L, is usually different from the FFT size, $N_{0}$, $P_{s}^{v,b}\left(m\right)$ in Equation (6) can be rewritten as:

(8) $$P_{s}^{v,b}\left(m\right)=e^{-j\pi 2{\tilde{d}}_{s}^{v,b}m/L}=e^{-j\pi 2\rho {\tilde{d}}_{s}^{v,b}m/N_{0}},\rho =N_{0}/L$$

where ρ denotes the ratio of $N_{0}$ to L and is a rational number. The non-integer factor, ρ, usually degrades the performance of the CBID detection. In order to compensate the effect of the fractional part in ρ on the CBID detection, we multiply $Z_{s}^{c,b}\left(m\right)$ by a polyphase sequence, $D_{s}^{v,b}\left(m\right)$, as follows:

(9) $$\begin{array}{l} {\tilde{Z}}_{s}^{c,b}\left(m\right)=Z_{s}^{c,b}\left(m\right)D_{s}^{v,b}\left(m\right)\\ =S_{s}^{c,b}Z_{s}^{g}\left(m\right)e^{-j\pi 2{\tilde{d}}_{s}^{v,b}m/N_{0}} \end{array}$$

where $D_{s}^{v,b}\left(m\right)=e^{-j\pi 2\Delta_{s}^{v,b}m/N_{0}},\Delta_{s,b}^{v}=\left(1-\rho \right){\tilde{d}}_{s}^{v,b}$.

In the following, $Z_{s}^{r,d}$, ${\tilde{X}}_{s}^{r,d}$, $X^{r,d}$, and $x^{r,d}$, used in Equations (2)–(4), are replaced by ${\tilde{Z}}_{s}^{c,b}$, ${\tilde{X}}_{s}^{c,b}$, $X^{c,b}$, and $x^{c,b}$, respectively, to formulate the equations for the CBID detection in mmWave cellular systems.

In addition, there may exist a residual STO even after the synchronization procedure has been completed with the SP. Because the residual STO causes a phase rotation in the frequency domain, the BP needs to be designed to be robust to the residual STO [16]. Next, we analyze the effect of the residual STO on the CBID detection. When the residual STO (χ) exists, the decimated version (even and odd samples) of $x^{c,b}\left({\left(n-\chi \right)}_{\%N}\right)$ with q∈{0,  1} is given by:

(10) $$\begin{array}{l} x^{c,b}\left({\left(2n+q-\chi \right)}_{\%N}\right)\\ =2/N\sum_{k=0}^{N_{0}-1}\left({\tilde{X}}_{s={\left(q+l\right)}_{\%2}}^{c,b}\left(k\right)e^{-j2\pi (u_{l}+l{\left(q+l\right)}_{\%2})k/N_{0}}\right)e^{j2\pi kn/N_{0}}\\ =2/NS_{s={\left(q+l\right)}_{\%2}}^{c,b}\sum_{m=0}^{L-1}\left(Z_{s={\left(q+l\right)}_{\%2}}^{g}\left(m\right)e^{-j\pi 2({\tilde{d}}_{s={\left(q+l\right)}_{\%2}}^{v,b}+u_{l}+l{\left(q+l\right)}_{\%2})\tilde{m}/N_{0}}\right)e^{j2\pi \tilde{m}n/N_{0}} \end{array}$$

where $\tilde{m}=N_{0}/2-\left\lfloor L/2\right\rfloor +m,\;\chi =2\mu_{l}+l$

$$U_{l}=\left\{u_{l}|\;0\leq u_{l}\leq \left\lfloor \chi_{Max}/2\right\rfloor -l{\left(\chi_{Max}+1\right)}_{\%2}\right\}$$

$$l={\left(\chi \right)}_{\%2},\;\chi \in \left\{0,1,\cdot \cdot \cdot ,\chi_{Max}\right\}$$

Here, l denotes a parameter indicating that χ is an even number (l=0) or an odd number (l=1). It can be seen from Equation (1) that $s={\left(q+l\right)}_{\%2}$ changes depending upon the value of the STO for the same q. When χ is an even number, the root index of the ZC sequence of a decimated version with q=0 in Equation (10) corresponds to the case, s=0. However, when χ is an odd number, the root index of a ZC sequence of a decimated version with q=0 corresponds to the case, s=1. Hence, an ambiguity in the GID detection may occur because the order of the indices in a root pair corresponding to the GID can be reversed depending upon the value of the STO in the CBID detection. For example, $N_{0}$-point FFTs of the even and odd samples of $x^{c,b}\left({\left(n-\chi \right)}_{\%N}\right)$ are expressed as ${\tilde{X}}_{0}^{c,b}\left({\left(k\right)}_{\%N_{0}}\right)e^{-j2\pi u_{0}k/N_{0}}$ and ${\tilde{X}}_{1}^{c,b}\left({\left(k\right)}_{\%N_{0}}\right)e^{-j2\pi u_{0}k/N_{0}}$, when χ is an even number, but as ${\tilde{X}}_{1}^{c,b}\left({\left(k\right)}_{\%N_{0}}\right)e^{-j2\pi (u_{1}+1)k/N_{0}}$ and ${\tilde{X}}_{0}^{c,b}\left({\left(k\right)}_{\%N_{0}}\right)e^{-j2\pi u_{1}k/N_{0}}$, when χ is an odd number. In order to avoid ambiguity, the value of ${\tilde{d}}_{0}^{v,b}$ needs to be selected such that it is not equal to ${\tilde{d}}_{1}^{v,b}$ in the preamble design. For example, if the value of ${\tilde{d}}_{0}^{v,b}$ is an odd number, then the value of ${\tilde{d}}_{1}^{v,b}$ needs to be an even number. Besides the ambiguity in the GID detection, ambiguities in detecting the SID and BID may occur because the effect of the STO is exhibited by a phase rotation in the frequency domain. For example, an ambiguity in the BID detection occurs when ${\left({\tilde{d}}_{s}^{v^{\prime },b^{\prime }}+u_{0}\right)}_{\%N_{0}}$ or ${\left({\tilde{d}}_{s}^{v^{\prime },b^{\prime }}+u_{1}\right)}_{\%N_{0}}$ is equal to ${\left({\tilde{d}}_{s}^{v^{\prime },b^{\prime \prime }\neq b^{\prime }}\right)}_{\%N_{0}}$; an ambiguity in the SID detection occurs when ${\left({\tilde{d}}_{s}^{v^{\prime },b^{\prime }}+u_{0}\right)}_{\%N_{0}}$ or ${\left({\tilde{d}}_{s}^{v^{\prime },b^{\prime }}+u_{1}\right)}_{\%N_{0}}$ is equal to ${\left({\tilde{d}}_{s}^{v^{\prime \prime }\neq v^{\prime },b^{\prime \prime }}\right)}_{\%N_{0}}$. Therefore, the value of ${\tilde{d}}_{s}^{v,b}$ should be selected such that ${\left({\tilde{d}}_{s}^{v^{\prime },b^{\prime }}+u_{0}\right)}_{\%N_{0}}\neq {\left({\tilde{d}}_{s}^{v^{\prime },b^{\prime \prime }\neq b^{\prime }}\right)}_{\%N_{0}}$ and ${\left({\tilde{d}}_{s}^{v^{\prime },b^{\prime }}+u_{0}\right)}_{\%N_{0}}\neq {\left({\tilde{d}}_{s}^{v^{\prime \prime }\neq v^{\prime },b^{\prime \prime }}\right)}_{\%N_{0}}$ because $U_{1}\subseteq U_{0}$.

Next, we consider an OFDM-based mmWave cellular system with a Tx-Rx beamforming. When the proposed preamble with the CID, c, and the BID, b, is transmitted from the b-th Tx beam of the BS, the received signal at the i-th Rx beam in the MS is given in the frequency domain as follows:

(11) $$Y_{i}(k)=\sum_{c=0}^{N_{C}-1}\sum_{b=0}^{N_{B}-1}G_{i}^{c,b}\left(k\right)\;X^{c,b}\left(k\right)e^{-j2\pi \chi^{c,b}k/N}+W_{i}\left(k\right)$$

where $G_{i}^{c,b}(k)=\eta^{c,b}\mu^{c,b}H_{i}^{c,b}(k)$.

Here, it is assumed that the initial STO and CFO synchronizations have been completed and only the residual STO exists. The parameters η, μ, H, G, and W denote the Tx array gain, Rx array gain, channel frequency response, total gain including the Tx and Rx array gains, and the additive white Gaussian noise (AWGN), respectively. The detection of the CID, BID, and the residual STO using the received signal can be performed by correlating the received signal in Equation (11) with the preamble sequence in the frequency domain as follows:

(12) $$\{\hat{c}=N_{v}\hat{g}+\hat{v},\hat{b},\hat{\chi }=2{\hat{u}}_{\hat{l}}+\hat{l},\hat{i}\}=\underset{g,v,b,l,u_{l},i}{\mathrm{arg max}}\left\{\Lambda_{l,u_{l},i}^{g,v,b}\right\}$$

(13) $$\Lambda_{l,u_{l},i}^{g,v,b}={\left|\sum_{q=0}^{1}\Omega_{s={\left(q+l\right)}_{\%2},i}^{g,q}\left({\tilde{d}}_{s={\left(q+l\right)}_{\%2}}^{v,b}+u_{l}+l{\left(q+l\right)}_{\%2}\right)\right|}^{2}$$

(14) $$\Omega_{s,i}^{g,q}\left({\left(n\right)}_{\%N_{0}}\right)=\;\sum_{m=0}^{L-1}{\tilde{Y}}_{i}^{q}\left(m\right){\left(Z_{s}^{g}\left(m\right)\right)}^{*}e^{j2\pi mn/N_{0}}$$

where ${\tilde{Y}}_{i}^{q}\left(m\right)=Y_{i}^{q}\left({\left(m+N_{0}/2-\left\lfloor L/2\right\rfloor \right)}_{\%N_{0}}\right)$

$$Y_{i}^{q}\left({\left(k\right)}_{\%N_{0}}\right)=\sum_{n=0}^{N_{0}-1}y_{i}\left(2n+q\right)e^{-j2\pi kn/N_{0}}$$

Here, $\hat{g}$, $\hat{v}$, $\hat{c}$, and $\hat{i}$ denote the detected GID, SID, CID, and Rx beam index, respectively. $\hat{\chi }$, $\hat{l}$, and ${\hat{u}}_{\hat{l}}$, denote the estimated parameters for the residual STO. $Y_{i}^{q}\left({\left(k\right)}_{\%N_{0}}\right)$ represents the frequency domain version of $y_{i}\left(2n+q\right)$. Here, $y_{i}\left(2n+q\right)$ denotes the decimated version of the received signal in the time domain with q∈{0,  1}. Because $r_{s}^{g}\in \left\{1,2,\cdot \cdot \cdot ,N_{r}\right\}$ is always satisfied for the root indices in $\left\{r_{0}^{g},r_{1}^{g}\right\}$ selected by Equation (7), the IFFT operation required for the CBID detection needs to be performed $N_{r}$ times for each ${\tilde{Y}}_{i}^{0}\left(m\right)$ and ${\tilde{Y}}_{i}^{1}\left(m\right)$. Therefore, the computational complexity for the CBID detection decreases as $N_{r}$ decreases.

## 4. Simulation

In this section, the performance of the proposed preamble design technique is evaluated by computer simulation with a simple setup of an OFDM-based mmWave cellular system, as shown in Figure 2. The center frequency, bandwidth, FFT size, CP size, and subcarrier spacing are set to 28 GHz, 250 MHz, 1024, 128, and 270 kHz, respectively [17]. A Rician channel model consisting of one LOS path and one NLOS path is used for an mmWave channel [3] and is programmed with a 3-dimensional spatial channel model (3D-SCM) [18]. The number of rays in the NLOS path is set to 20, and the K-factor is set to 10 dB in our simulation.

In the simulation, only BS1 is considered for a one-cell environment and both BS1 and BS 2 are considered for a two-cell environment. The cell radius is set to 500 m. In a two-cell environment, it is assumed that the MS with a switched beam is located 400 m away from BS1. The residual STO (χ) is generated uniformly in the range 0–4. A uniform linear array (ULA) with eight antenna elements is used at the BS. A uniform circular array (UCA) with eight antenna elements is used at the MS to cover a 360° azimuth angle around the MS. The parameters for the preamble generation, beamforming, and the CBID information used in the simulation are listed in Table 2.

**Figure 4:**
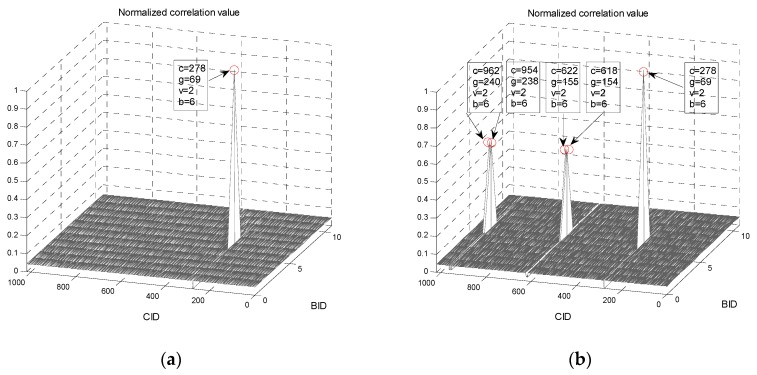
Correlation properties of the proposed preamble depending on the value of $N_{r}$: (**a**) Type-1; (**b**) Type-2.

Different notations are used for the length of ZC sequence, L, to distinguish between the parameters used for each technique. The notation, $\bar{L}$, is used for the previous technique [9] and $\tilde{L}$ is used for the proposed technique. Here, the previous technique refers to the case where the CBID information is mapped to the preamble sequence generated by a single ZC sequence. In the proposed technique, a preamble sequence is generated by a combination of two ZC sequences. Here, $\bar{L}$ and $2\tilde{L}$ are set to 1021 and 1018, respectively. The number of CIDs, $N_{C}$, is set to 1020 ($\bar{L}-1$) for both the previous and the proposed techniques. The values of $L_{P}$ and $\left\{O_{0}^{v},O_{1}^{v}\right\}$ are selected such that ${\tilde{d}}_{s}^{v,b}$ satisfies the conditions described in Section 3 and are listed in Table 2. In the simulation, two types of the proposed preamble sequences (Type-1 and Type-2) with different values of $N_{r}$ are considered. The value of $N_{r}$ is set to 255 for Type-1 and 86 for Type-2, respectively. The value of $N_{g}$ is set to 255 for both Type-1 and Type-2. Figure 4 shows the correlation properties of the proposed preamble sequences (Type-1 and Type-2) when CID is 278 and BID is six. It can be seen from Figure 4a that the preamble sequence of Type-1 has a good correlation property because the number of side-peaks is zero. Note that the number of side-peaks (satisfying the condition in Property 3) is given by 2(w−1), where $w=\left\lceil N_{g}/N_{r}\right\rceil$. In the case of the Type-2 preamble, there are side-peaks with a value of 0.5 at four different CIDs because the value of 2(w−1) is four, as can be seen in Figure 4b. The root index pairs corresponding to the CIDs (618, 622, 954, 962) are ({69, 70}, {70, 71}, {68, 70}, {70, 72}), whereas the root index pair corresponding to the CID (278) is {70,70}. A high correlation occurs at these four CIDs with BID = 6 because the same root index, 70, is used.

**Figure 5:**
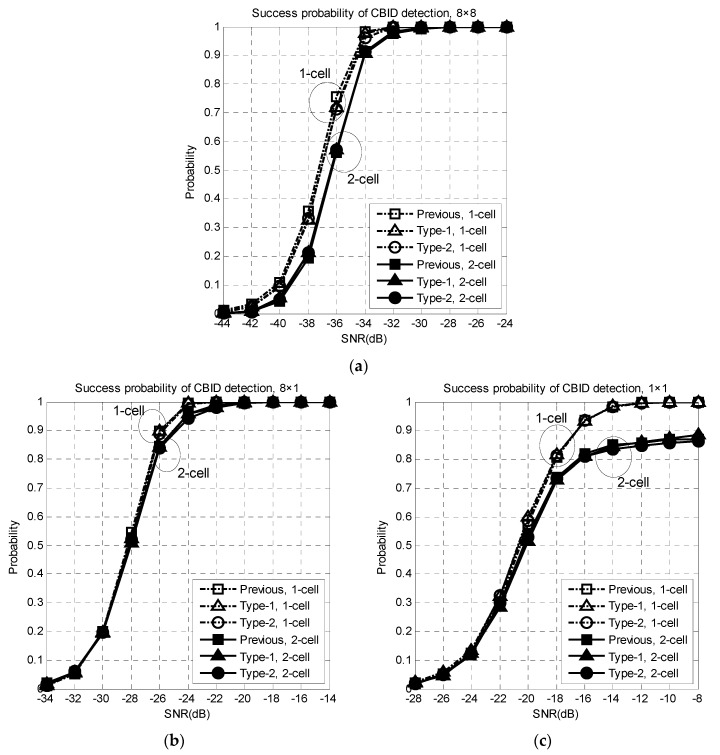
Success probability of the CBID detection in one-cell and two-cell environments: (**a**) 8 × 8; (**b**) 8 × 1; (**c**) 8 × 1.

Figure 5 shows the success probability of the CBID detection in one-cell and two-cell environments. CBID detection using the preambles simultaneously transmitted from the BS(s), is performed at the MS using Equations (12)–(14). The success of the CBID detection refers to the case where the CID and BID detected at the MS match the ones used in the BS for the beam transmission.

Simulations are performed for three different cases (1 × 1, 8 × 1, 8 × 8), where the first and second terms denote the number of antenna elements in the BS and MS, respectively. When the number of antenna elements is equal to one, an omnidirectional antenna is used. From this figure, it can be seen that an array gain of 9 dB is obtained when the number of antenna elements increases from one to eight. The success probability of the CBID detection decreases in a two-cell environment compared to a one-cell environment because of the interference transmitted from the adjacent BS. It can be seen from this figure that the proposed techniques (both Type-1 and Type-2) are robust to the STO and achieve almost the same performance as the previous technique. The reason that the proposed technique, Type-2, does not suffer from a performance degradation is that the parameters that can avoid the condition in Property 3 and ambiguities in the (GID, SID, BID) detection are selected at the cell planning stage. In the simulation, the CIDs of BS1 and BS2 are selected as 278 and 378. The root index pairs corresponding to the CIDs are {70, 70} and {9, 10}, respectively, where no common root index is used. The performance degradation caused by the intercell interference (high correlation) in the Type-2 preamble can be avoided by selecting appropriate root index pairs at the cell planning stage. Note that the computational complexity required for the CBID detection in the Type-2 preamble is reduced to 33.3%, compared to the Type-1 preamble because $N_{r}$ in the Type-2 preamble is 1/3 that of the Type-1 preamble.

**Figure 6:**
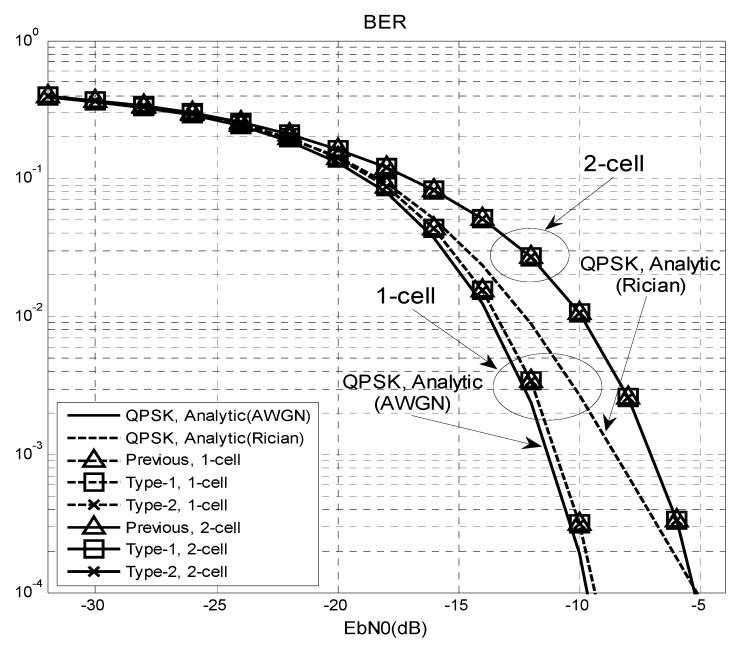
BER performance in one-cell and two-cell environments.

Figure 6 shows the bit error rate (BER) performance of an 8 × 8 mmWave system in Figure 2. The BER is measured during the data transmission period after completing the cell and beam search period. A quadrature phase shift keying (QPSK) signal is used for the input and the channel impulse response is assumed to be known. In this figure, analytic curves for AWGN and Rician channel (k-factor = 10 dB) with an 8 × 8 beamforming are included for comparison. From this figure, it can be seen that the BER performance of the beamforming system (one-cell) is better than the analytic (Rician) curve because the channel can be approximated as an AWGN when beamforming is performed in the direction of an LoS path. The performance is slightly worse than the AWGN case because a small power transmitted through the non-LOS path of the Rician channel will not be received in the MS. The previous and proposed (Type-1 and Type-2) techniques exhibit the same performance because the success probability of the CBID detection is one in the range of SNR, >−30 dB, as shown Figure 5. In a one-cell environment, the BER performance of the proposed (or previous) technique with an 8 × 8 beamforming is similar to the AWGN case. In a two-cell environment, the BER performance is degraded by approximately 4 dB compared to the one-cell case because of the interference from the adjacent BS.

**Figure 7:**
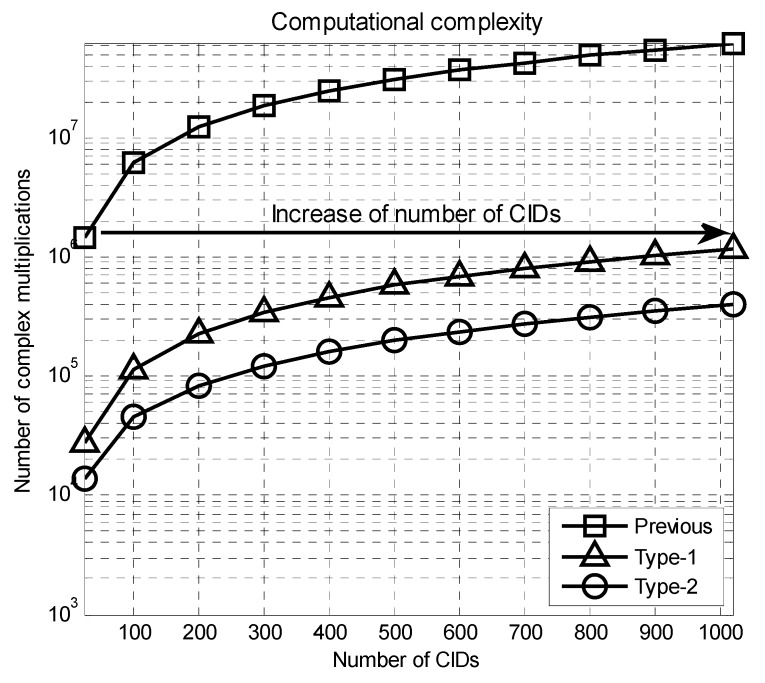
Number of complex multiplications required for the CBID detection when $N_{C}$ varies.

Table 3 shows the number of complex multiplications required for CBID detection at the MS for each Rx beam direction. From this table, it can be seen that the numbers of complex multiplications required for the Type-1 and Type-2 preambles are reduced to 1.9% and 0.6%, respectively, compared to the previous technique, when $N_{C}$ is 1020. A significant reduction in the computational complexity can be achieved using the Type-2 preamble. Figure 7 shows the number of complex multiplications required for the CBID detection when $N_{C}$ varies. When $N_{C}$ is 1020, the number of multiplications required for the Type-1 and Type-2 preambles are 1,175,040 and 396,288, respectively. When $N_{C}$ is 24, the number of multiplications required for the previous technique is 1,470,240 that is larger than that for the proposed technique (Type-1 and Type-2), when $N_{C}$ is equal to 1020. Therefore, the proposed technique (Type-1 and Type-2) can provide an increase in the number of CIDs by a factor of 43, compared to the previous technique, when the same number of complex multiplications is used for the CBID detection.

## 5. Conclusions

In this paper, an efficient preamble design technique is proposed for OFDM-based mmWave cellular systems with beamforming, by combining two ZC sequences in the frequency domain. The parameter selection methods for the proposed preamble are also described for avoiding the performance degradation caused by the side-peaks (high correlation), STO, and ambiguities in the (GID, SID, BID). It was shown by simulation that the proposed techniques (Type-1 and Type-2) can obtain a significant complexity reduction in the CBID detection without a noticeable performance degradation compared to the previous technique. The number of complex multiplications required for the Type-2 preamble is reduced to 0.6% compared to the previous technique. The performance degradation caused by the side-peaks in the Type-2 preamble can be avoided by selecting appropriate root index pairs in the cell planning stage. With a similar computational complexity for the CBID detection, the proposed technique can provide an increase in the number of CIDs by a factor of 43, compared to the previous technique.

## Figures and Tables

**Table:** (**a**)

g	$r_{0}^{g}$	$r_{1}^{g}$	g	$r_{0}^{g}$	$r_{1}^{g}$
0	1	1	6	7	7
1	2	2	7	8	8
2	3	3	8	9	9
3	4	4	9	10	10
4	5	5	10	11	11
5	6	6	11	12	12

**Table:** (**b**)

g	$r_{0}^{g}$	$r_{1}^{g}$	g	$r_{0}^{g}$	$r_{1}^{g}$
0	1	1	6	7	7
1	2	2	7	1	2
2	3	3	8	2	3
3	4	4	9	3	4
4	5	5	10	4	5
5	6	6	11	5	6

**Table:** (**c**)

g	$r_{0}^{g}$	$r_{1}^{g}$	g	$r_{0}^{g}$	$r_{1}^{g}$
0	1	1	6	2	3
1	2	2	7	3	4
2	3	3	8	4	5
3	4	4	9	1	3
4	5	5	10	2	4
5	1	2	11	3	5

**Table 2:** Parameters for the simulation.

**Parameters for Preamble Generation**						
$\left(\tilde{L},\;L_{P}\right)$			(509,3)			
$\left(N_{C},\;N_{g},\;N_{v},\;N_{B},\;N_{M}\right)$			(1020,255,4,12,12)			
$N_{r}$			Type-1		Type-2	
			255		86	
b			0~11			
$\left\{O_{0}^{v},O_{1}^{v}\right\}$			{0,256},{36,292},{72,328},{108,36}			
$\left(N,\;N_{CP}\right)$			(1024,256)			
**Parameters for Beamforming**						
The number of antenna elements at BS and MS			8			
Antenna spacing			λ/2			
Steering factor of *m*-th antenna at ULA			$e^{j\pi \cos\left(\phi \right)\sin\left(\theta \right)m}$, *m* = 0~7			
Set of predefined angles (degree) at ULA (ϕ = azimuth angle, θ = elevation angle)			ϕ = {−55, −45, −35, −25, −15, −5, 5, 15 ,25, 35, 45, 55}, θ = 0 (fixed)			
Steering factor of *m*-th antenna at UCA			$e^{j\pi \sin\left(\theta \right)\sin\left(\phi -2\pi m/8\right)m}$, *m* = 0~7			
Set of predefined angles (degree) at UCA (ϕ = azimuth angle, θ = elevation angle)			ϕ = {0, 30, 60, 90, 120, 150, 180, −150, −120, −90, −60, −30}, θ = 0 (fixed)			
AOD (Line-of-sight)			BS1: 5°/BS2: −5°			
AOA (Line-of-sight)			MS: 0°/MS: 180°			
AOD (Non-line-of-sight)			BS1: −25°/BS2: 25°			
AOD (Non-line-of-sight)			MS: 30°/MS: 150°			
**CBID Information**						
	GID		SID	CID		BID
	Type-1	Type-2				
BS1	69/{70,70}	69/{70,70}	2	278		6
BS2	155/{28,29}	155/{70,71}	2	622		0~11

**Table 3:** Number of complex multiplications required for the CBID detection.

	The Number of Complex Multiplications		Example
Previous technique	$N_{C}N_{B}\bar{L}(\chi_{max}+1)$		62,485,200
Proposed technique	$N_{r}(N/2)\times \log_{2}(N/2)$	Type-1 ($N_{r}=255$)	1,175,040
		Type-2 ($N_{r}=86$)	396,288
